# Airway management procedures in Swedish emergency department patients - a national retrospective study

**DOI:** 10.1186/s12873-022-00627-3

**Published:** 2022-04-21

**Authors:** Susanne B. Wilhelms, Daniel B. Wilhelms

**Affiliations:** 1 Department of Anaesthesia and Intensive Care in Linköping, Linköping, Sweden; 2 Department of Biomedical and Clinical Sciences, Linköping University, Linköping, Sweden; 3 Department of Clinical Physiology in Linköping, Linköping, Sweden; 4 Department of Medical and Health Sciences, Linköping University, Linköping, Sweden; 5 Department of Emergency Medicine in Linköping, Linköping, Sweden

**Keywords:** Airway management, Endotracheal intubation, Emergency department, Epidemiology

## Abstract

**Background:**

With the on-going debate about which specialty should be responsible for intubations in the emergency department in mind, the aim of this study was to describe the prevalence of endotracheal intubation and other airway management procedures in emergency department patients in Sweden.

**Methods:**

All patients registered in the Swedish Intensive Care Registry with admission date from January 1 2013 until June 7 2018 and reported admission type “from the emergency department” or “emergency department” reported in the SAPS3 scoring were included. All patients missing codes for procedures were excluded.

**Results:**

A total of 110,072 admissions from an emergency department to an ICU were registered during the study period. Of these, 41,619 admissions (37.8%) were excluded due to lack of codes for medical procedures. The remaining 68,453 admissions (62.2%) were included, and 31,888 emergency airway procedures (within 3 h from admission time to the intensive care unit) were registered. Invasive emergency airway procedures were the most common type of airway procedure (*n* = 23,446), followed by non-invasive airway procedures (*n* = 8377) and high-flow nasal cannula (*n* = 880). In 2017 a total of 4720 invasive emergency airway management procedures were registered.

**Conclusions:**

The frequency of invasive airway management procedures in Swedish EDs is low. With approximately 1.9 million adult ED visits per year, this gives an estimated incidence of 2.4 invasive airway management procedures per thousand ED visits in 2017.

**Trial registration:**

Not applicable.

## Background

Endotracheal intubation is a high-risk procedure in critically ill patients, and complications such as severe hypoxemia and hemodynamic collapse are relatively common [1]. It is obvious that training is a key to successful intubation, both for obtaining and maintaining the skill. Therefore, it is necessary to assure that staff performing emergency airway management procedures have the opportunity to get sufficient training to gain and maintain their competence.

Generally, there is no easy answer on how to gain and maintain competence in airway management. For instance, there is no consensus on how many endotracheal intubations one should perform to gain sufficient experience for intubation in non-elective patients [2]. Some suggest that < 40 intubations are enough to gain proficiency [3], whereas other studies indicate that 200 endotracheal intubations are needed to gain enough experience for safe endotracheal intubation in emergency situations [4]. Special circumstances may require even more training, as indicated by a study by Kim et al., in which 240 intubations s were required to gain a high rate of successful intubations in cardiopulmonary resuscitation situations [5].

The literature on maintaining endotracheal intubation skills is sparse, and there is no clear evidence on how many intubations are needed on a yearly basis to maintain good endotracheal intubation skills. However, the problem with lack of intubation opportunities after the initial intubation training phase is well-described. In a study by Carlson et al., covering 135 emergency departments in the U.S., the intubation incidence per ED physician varied considerably between 0 and 109 intubations per year (median 10) [6], with 24% of the emergency physicians performing fewer than 5 intubations per year. The problem with few intubation opportunities is also described for other professions, such as respiratory therapists and paramedics [7, 8].

One way of assessing competence is working with “entrustable professional activites” (EPA), where trainees are systematically assessed by supervisors in specific activities, such as emergency airway management. For example, rapid sequence induction (a key skill for performing successful emergency airway management) is a common EPA for residents in anesthesia. However, in a Swiss study, a majority of supervisors did not think rapid sequence induction should be performed un-supervised within the first year of training in anesthesiology [9]. This would either indicate that rapid sequence induction (and, as a consequence, emergency airway management) is an EPA which needs long-term training to achieve, or that the number of training opportunities is low.

Traditionally, the most common specialties performing airway management in ED patients are emergency medicine physicians, anesthesiologists and critical care physicians. In many European countries, including Sweden, most endotracheal intubations are performed by anesthesiologists. However, with the rapid growth of emergency medicine as a specialty in Europe there is an on-going discussion, with differing opinions, on what specialty, i.e. anesthesiologists/intensivist or ED physicians, should be responsible for such procedures in the emergency department [10–15].

However, the crucial perspective in this discussion might not actually be what specialty should do the tube. Instead, it is important to ensure that all physicians who perform emergency airway management have sufficient opportunities to gain and maintain their skills in airway management so that it is performed safely and skillfully when need arises. To ensure this, it is crucial to know the prevalence of such procedures in ED patients. Without European counterparts to the US National Emergency Airway Registry (NEAR) or the Australian and New Zealand Emergency Department Airway Registry (ANZEDAR) [16, 17], there is, however, no simple way to obtain an overview of ED airway management in Europe. The NAP4 study in United Kingdom (UK) included a praiseworthy description of complications in airway management in emergency departments and ICUs, but it was limited in coverage to not more than about 50% of the emergency departments in UK and might only have seen the “tip of the iceberg” [18]. In addition, the focus of the NAP4 study was not the incidence of normal non-complicated airway management procedures or opportunities to gain and maintain airway skills.

Even with robust data from studies derived from existing airway registers such as NEAR (United States) and ANZEDAR (Australia and New Zealand) [16, 17], it is important to also describe the situation in European and Scandinavian EDs, since factors related to culture, education and hospital organization clearly influence where and how airway management procedures are performed. Sweden lacks a specific register for airway procedures, but has a well-established Swedish Intensive Care Registry (SIR), covering admissions to about 90% of all ICUs in Sweden. Since all intubated patients are admitted to an ICU for further care, these data provide an opportunity to study the prevalence of emergency airway management procedures in Sweden.

With the on-going debate about which specialty should be responsible for intubations in the emergency department in mind, the aim of this study was therefore to describe the prevalence of endotracheal intubation and other airway management procedures in emergency department patients in Sweden.

## Methods

All patients registered in the Swedish intensive care Registry with admission date from January 1, 2013 until June 7, 2018 and reported admission type “from the emergency department” or “emergency department” reported in the Simplified Acute Physiology Score 3 (SAPS3) scoring were included. All patients missing codes for procedures were excluded. These criteria for inclusion and exclusion were defined a priori, and a data manager at Swedish intensive care Registry extracted requested data from the registry, including individual patient number, age, gender, admission and discharge date, hospital type (academic, community, rural), primary ICU diagnosis, secondary ICU diagnoses, procedures, SAPS3 scoring and outcome (dead, alive). All further analyses and sub-grouping were done by us.

Airway procedures are coded as medical procedures in SIR [19], and we extracted the following: start of acute treatment with continuous positive airway pressure (CPAP) or bilevelPAP (Swedish classification of medical procedures code: DG001), start of mechanical ventilator treatment (DG002), supplemental oxygen (DG015), endotracheal intubation (DG017), endotracheal intubation with fiber endoscopy (DG018), percutaneous tracheotomy (DG020), conventional treatment with mechanical ventilator (DG021), high-frequency oscillation mechanical ventilator (DG022), non-invasive treatment with mechanical ventilator (DG023), coniotomy (DG025), treatment with mechanical ventilator more than 96 h (DG026), start of long-time treatment with CPAP and Bilevel PAP (DG027), supplemental oxygen with high-flow nasal cannula (DG028), tracheostomy (GBB00), percutaneous tracheostomy (GBB03) and other tracheostomy surgery (GBB96). These procedures were grouped in the following groups: invasive airway management (DG002, DG017, DG018, DG020, DG021, DG022, DG025, DG026, GBB00, GBB03, GBB96), non-invasive airway management (DG001, DG023, DG027) and supplemental oxygen with high-flow nasal cannula (DG028).

All airway management procedures registered within 3 h from admission time point to the intensive care unit were considered as emergency procedures. Sub-grouping within 0, 1, 2 and 3 h were done to evaluate the degree of urgency.

The main outcome was prevalence of invasive airway management procedures within 3 h from admission time to the intensive care unit, with other airway management procedures analyzed as secondary outcomes.

The study protocol was approved by the Regional Ethical Review Committee in Linköping, Sweden on 18 April 2018 (2018/177–31) and a supplement to the study protocol was approved by the Swedish Ethical Review Authority on 21 November 2021 (2021–05648-02), and the requirement for informed consent was waived. All methods were performed in accordance with the Declaration of Helsinki and relevant Swedish regulations.

### Statistics

All data were analyzed in STATA version 14 (StataCorp, College Station, TX, USA). Demographic data were reported as mean (standard deviation). Groups of patients were compared using the Student’s t-test (continuous data) and the chi-square test (categorical data). Differences were considered statistically significant at a *p*-value of < 0.05.

## Results

A total of 80 ICUs, in 61 hospitals, reported data during the study period. A total of 110,072 admissions from an emergency department to an ICU were registered during the study period. Of these, 41,619 admissions (37.8%) were excluded due to lack of codes for medical procedures. The remaining 68,453 admissions (62.2%) were further analyzed.

The excluded patients were generally younger, had lower SAPS3 score at admission, had shorter length of stay in the ICU and lower mortality rates compared to the included patients (Table 1). It was also more common with admission for only observation (according to the SAPS3 scoring) in the excluded group. A higher proportion of patients was excluded in rural hospitals, where a majority of patients missed codes for medical procedures.

**Table 1:** Demographics for included and excluded patients

	Included	Excluded (no medical procedure)	p
**Age**	56.2 ± 0.8 SD	50.0 ± 0.12 SD	< 0.01
**Female sex (%)**	42.2%	42.9%	0.02
**SAPS3**	55.9 ± 0.06 SD	45.1 ± 0.06 SD	< 0.01
**Only observation (SAPS 3 at admission)**	5.7%	23.7%	< 0.01
**Length of stay in ICU (days)**	2.5 ± 0.02 SD	0.8 ± 0.004 SD	< 0.01
**Mortality**	10.4%	1.9%	< 0.01
**Rural hospital**	46.5% (*n* = 16,156)	53.5% (*n* = 18,579)	
**Community hospital**	64.0% (*n* = 32,606)	36.0% (*n* = 18,366)	
**Academic hospital**	80.8% (*n* = 19,691)	19.2% (*n* = 4674)	

Of the admissions with registered codes for medical procedures (*n* = 68,453), a majority (*n* = 38,110) had a code for airway management-related procedures. Most of the airway management procedures (*n* = 31,888, 83.7%) were performed within 3 h from admission to the ICU, i.e. emergency airway management procedures.

The most common type of emergency airway management in this population was invasive airway management (*n* = 23,446, 71.3% of the emergency airway management procedures) (Table 2). The second most common was non-invasive airway management (*n* = 8377, 25.5%), whereas oxygen supplement with high-flow nasal cannula was less common (*n* = 880, 2.7%).

**Table 2:** Emergency airway management procedures in all included patients

	Invasive	Non-invasive	High-flow nasal cannula
**All**	34.3% (*n* = 23,446)	12.2% (*n* = 8377)	1.3% (*n* = 880)
**Rural hospital**	18.9% (*n* = 3056)	15.3% (*n* = 2470)	1.2% (*n* = 193)
**Community hospital**	34.1% (*n* = 11,101)	12.9% (*n* = 4215)	0.8% (*n* = 272)
**Academic hospital**	47.2% (*n* = 9289)	8.6% (*n* = 1692)	2.1% (*n* = 415)

Invasive emergency airway management was more prevalent in academic ICUs (*n* = 9289, 47.2% of admissions with registered medical procedures codes), compared to community (*n* = 11,101, 34.1%) and rural ICUs (*n* = 3056, 18.9%).

**Fig. 1:**
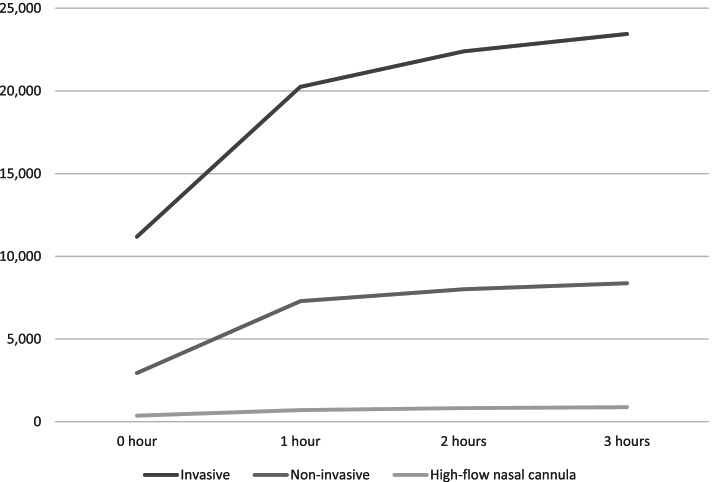
Time (from ICU admission) of emergency airway management procedures

A majority of the emergency airway management procedures were performed either before or at the moment of ICU admission (hour: 0) or within the first hour of ICU admission (hour: 1) (Fig. 1).

Over the years studied, invasive airway management procedures and oxygen therapy with high-flow nasal cannula increased whereas noninvasive airway management procedures did not increase (Table 3).

**Table 3:** Emergency airway procedures in relation to year performed

Year	Invasive	Non-invasive	High-flow nasal cannula
	n, (% of all admissions with a code for a medical procedure)		
**2013**	3934 (32.2%)	1493 (12.2%)	2 (0.02%)
**2014**	4099 (32.4%)	1506 (11.9%)	8 (0.06%)
**2015**	4262 (33.8%)	1580 (12.5%)	110 (0.87%)
**2016**	4559 (35.9%)	1536 (12.1%)	208 (1.6%)
**2017**	4720 (35.7%)	1619 (12.2%)	332 (2.5%)

## Discussion

During the 5.5 years studied, 31,888 emergency airway management procedures were registered in ICU patients admitted from the emergency department. The most prevalent type of emergency airway management procedures were invasive ones, reaching 4720 procedures in 2017. In relation to the number of emergency department visits in Sweden yearly (1.9 million adult visits in year 2017 [20]) the estimated incidence of invasive airway management procedures would be 2.4/1000 ED visits.

To make sense, the number of procedures also needs to be related to staffing levels in Swedish emergency departments. However, Swedish EDs are staffed in different ways; either by emergency physicians (and trainees), or by rotating physicians from other departments in the hospital (for example medicine, surgery, orthopedics, neurology, infection), or by a combination of emergency physicians and rotating physicians. Therefore, it is difficult to estimate staffing levels in a national perspective [21].

However, examples from EDs of different sizes can give some indication of the order of magnitude of invasive airway procedure in relation to staffing. In Östergötland county with 467,000 inhabitants there are two hospitals with ICUs (Linköping and Norrköping). The ED in Linköping (academic hospital) is only staffed with emergency physicians and trainees, a total of about 60 physicians. In 2017 the number of invasive emergency airway management procedures were 124, resulting in approximately 2 intubations/physician and year. In a rural hospital in Skåne county, Ystad hospital, mainly staffed by emergency physicians and trainees, the number of invasive emergency airway management procedures in 2017 was 28, resulting in approximately 1.75 intubations/physician and year. A system with rotating physicians from other departements would probably result in more individuals working in the ED, and as a consequence even less intubations/physician and year. With previous literature about number of intubations needed to gain profiency in emergency situations in mind, this is a very low number.

Another important aspect when discussing airway management and training opportunities in the ED is prehospital intubations. There is no recent study on the incidence of prehospital intubations in Sweden. However, in our county Östergötland, the incidence of prehospital intubations is low. In 2017 the ambulance service registered 30 endotracheal intubations (personal communication), compared to 204 invasive emergency airway management procedures in hospital (Linköping and Norrköping combined). The total number of ambulance missions in the county in 2017 was approximately 48,500, rendering an incidence of 0.62 intubtions/1000 amublance missions, the majority of which were performed in CPR situations [20]. As mentioned in the introduction, the problem with few endotracheal intubation procedures per year and individual is also well-described in other countries and for other professions [6–8], especially in the pre-hospital setting. Cobas et al. has showed 31% failed prehospital intubations in a prehospital setting in the U. S [22]. It has been discussed whether this high rate of failed intubations is related to a dilution of intubation skills, with too few procedures per paramedic each year [23]. On the contrary, a success rate of 100% of prehospital intubations has been described by Helm et al. in a setting of helicopter emergency medical service where experienced trauma anesthetists performed the intubations [24]. The circumstances prehospitally may differ significantly from an in-hospital emergency department, and it is unclear to which extent the results from pre-hospital studies may be generalizable to other settings. Not surprisingly, however, the general tendency in the literature is that the success rate of emergency airway management procedures seems to increase with experience.

The problem with few endotracheal intubation procedures per individual may in part be overcome by simulation training. However, a systematic review reports that simulation training is not superior to non-simulation training (lectures, videos, self studies, problem-based learning and clinical observation) [25]. The challenges associated with few endotracheal intubation procedures is frequently discussed also for other specialties, such as trainees in pulmonary and critical care medicine. For trainees in these specialties, simulation and training in operating theatres may serve as a bridge to emergency airway management in the ICU – but it cannot fully compensate training opportunities in real-life emergency airway management [2]. Another aspect of invasive airway management is the technical development with the wide introduction of video-laryngoscopes. Video-laryngoscopes seem promising especially for intubations in patients with limited view (Cormace-Lehane grade III and IV) where video-laryngoscope seems to be superior to Macintoch/Miller blades [26]. In another study, video-laryngoscopes tended to increase the rate of first attempt success when compared to direct laryngoscopy [27]. It has also been shown that video-laryngoscopy (versus Macintosh blade) is faster and associated with less adverse events when used by unexperienced users (in simulation situations) [28]. In summary, video-laryngoscope should be available and used in the ED but one should also remember that video-laryngoscope does not solve all problems and in these cases it is important to have knowledge and experience of other methods for airway management.

A major limitation in our study is the large group of excluded patients due to lack of medical procedure codes. The reasons for lack of medical procedure codes are most likely multiple. When comparing the excluded patients with the included ones, the excluded patients seem to be less ill as indicated by lower SAPS3 score, shorter length of stay in the ICU and lower mortality rate. There was also a higher rate of patients who were only admitted for observation in the excluded group. In summary, many parameters indicate that the excluded group was healthier – and perhaps did not require any medical procedures. However, another explanation of missing codes for medical procedures could be inadequate registration of codes. This is a common problem in register-based research. The data in the Swedish intensive care register is reported prospectively during the ICU stay, and the registry has an automatic check for logical errors. In addition, local validations of diagnoses and procedures are done, but the registry has not been generally/externally validated. Some variables in the Swedish intensive registry, such as age, hospital type and primary diagnosis, are compulsory but medical procedures are not, which may also contribute to a higher frequency of missing data for procedures.

Another limitation may be the definition of “emergency” airway management procedures. Surveying previous literature on this topic, it is obvious that there is no consensus on how to define an emergency airway management procedure vs a non-emergent one. We have chosen to define an emergency procedure as a procedure performed within 3 h from the time of ICU admission. We believe that a 3 h time limit could be an appropriate cut-off to indicate which patients could potentially be eligible for airway management procedures in the emergency department, and we are aware that a patient’s condition could change dramatically within a 3 h time frame – i.e. a patient who is intubated after 2.5 h in the ICU might not have been appropriate to intubate already in the emergency department. Therefore, a time-limit of 3 h after ICU admission may result in an overestimation of emergency airway management procedures. However, a majority of the procedures were performed before ICU admission or within the first hour in ICU (Fig. 1).

To overcome several of the limitations in this study and to facilitate in-depth studies of airway management procedures in an emergency setting, it would be valuable to institute a European version of the American National Emergency Airway Registry (NEAR). In the light of the current debate on emergency airway management, and before making any changes in the organization of emergency airway management procedures, we suggest the establishment of a quality register focusing on airway management procedures. This register should also include information about, for example, first-pass success, surgical airway procedures, the provider’s experience of intubation, as well as data on complications, none of which can be evaluated with the existing Swedish intensive care register. Another question we cannot answer with the existing data is the number of emergency endotracheal intubations per individual physician, nor the success rate of intubation attempts. There may also be regional differences resulting in more frequent emergency endotracheal intubations in specific emergency departments, even if the mean number of invasive airway management procedures per physician is low on a national level.

In summary, our study shows that the number of invasive airway management procedures in Swedish EDs is low. This needs to be considered when implementing strategies for emergency airway management, since gaining and maintaining competence is a key factor for successful intubation in emergency situations. However, local conditions may vary, and it is important to take these into consideration when discussing the generalizability of our results to other countries and health care systems.

## Conclusion

The number of invasive airway management procedures is low in Swedish emergency department patients, indicating that training and maintaining proficiency in acute intubations is likely to be challenging in most emergency departments. Based on these findings, there is a clear need for better data regarding airway management procedures in the emergency department, before implementing any changes in the organization of the care for these patients.

## Data Availability

The data that support the findings of this study are available from the Swedish intensive care registry but restrictions apply to the availability of these data, which were used under license for the current study, and so are not publicly available. Data are however available from the authors upon reasonable request and with permission of the Swedish intensive care registry.

## Abbreviations

ED: Emergency department
ICU: Intensive care unit
SAPS3: Simplified acute physiology score 3
